# Assessing the Prevalence and Risk Factors of Postpartum Depression: A Cross-Sectional Study Conducted in the Urban Areas of Ankuli in Southern Odisha

**DOI:** 10.7759/cureus.61503

**Published:** 2024-06-01

**Authors:** Madhumita Bhakta, Durga M Satapathy, Manisha Padhy, Sithal Dalai, Jasmin N Panda, Pramila Marandi, Swamy SVN, Amita Pattnaik

**Affiliations:** 1 Community Medicine, Maharaja Krushna Chandra Gajapati (MKCG) Medical College and Hospital, Brahmapur, IND

**Keywords:** mother, screening, risk factors, postpartum depression, epds

## Abstract

Background

Postpartum depression (PPD) is a complex mix of physical, emotional, and behavioral changes that happen in some women after giving birth.

Objectives

The aim of the study is to determine the prevalence of PPD using the Edinburgh Postnatal Depression Scale (EPDS) and evaluate the predisposing factors for PPD.

Methodology

The present observational study was conducted in the Department of Community Medicine, Maharaja Krushna Chandra Gajapati (MKCG) Medical College and Hospital, Brahmapur, Odisha, India from May 2022 to November 2022. Using the EPDS, participants were assessed for postnatal depression. Every subject additionally filled out a risk factor questionnaire covering important sociodemographic and obstetric parameters. The prevalence of an EPDS score of 12 or above is the primary outcome measure.

Results

The study encompassed 121 mothers, with 8.26% scoring above the depression cutoff of 12 and 6.61% falling within the borderline range. Notably, all mothers surpassing the cutoff were from joint families, contrasting with those from nuclear families. A predominant portion of the depressive group was in their 20s, while the borderline group primarily consisted of mothers in their 30s. Urban residency and government hospital care were universal among the samples. Mode of delivery showed significance, with a higher prevalence of PPD observed among those who underwent a lower segment cesarean section. Additionally, maternal age, anemia, mode of delivery, educational status, adverse life events, and lack of partner support significantly correlated with depression scores. Notably, maternal age emerged as the most influential factor, followed by anemia and mode of delivery. Spearman correlation analysis revealed moderate negative associations between various aspects of maternal depression and the ages of their babies, indicating that younger infants were associated with greater maternal distress. However, the correlation between feeling sad or miserable and the baby’s age was negligible. These findings emphasize the multifaceted nature of PPD, highlighting the interplay between sociodemographic factors, maternal well-being, and infant age.

## Introduction

The postpartum phase, spanning from the moment of birth to 42 days thereafter, is a crucial time for all women experiencing physical, emotional, and psychological changes. The exhaustion of labor and delivery, the excitement of bringing forth a new life, and its demands affect the mental health of all mothers [1]. India has made significant strides in improving maternal and child health outcomes through initiatives such as the Reproductive, Maternal, Newborn, Child, and Adolescent Health (RMNCHA+) strategy under the National Health Mission [2]. This comprehensive approach encompasses various horizontal programs. For instance, the Pradhan Mantri Surakshit Matritva Abhiyan strives to offer guaranteed, thorough, and high-quality prenatal care at no charge to all expectant mothers on the ninth of each month [3]. Mother’s Absolute Affection (MAA) prioritizes the promotion of breastfeeding and the provision of counseling services to bolster breastfeeding support within healthcare systems [4]. Surakshit Matritva Aashwasan (SUMAN) is dedicated to ensuring the dependable delivery of maternal and newborn healthcare services, emphasizing broader access to free and high-quality care while maintaining a policy of zero tolerance for service denials [5]. SUMAN also ensures effective management of complications and upholds the autonomy, dignity, feelings, choices, and preferences of women. Additionally, the Quality Improvement Initiative in Labour Room & Maternity OT (LaQshya) targets enhancing the quality of care provided to mothers and newborns during labor and immediately after childbirth [6].

Despite these efforts, postpartum depression (PPD) remains a significant public health concern, impacting the well-being of mothers and their infants. Recognizing the importance of addressing PPD, WHO has recommended integrating mental health services into existing maternal health programs and promoting awareness about PPD among healthcare providers and communities. Additionally, tackling PPD aligns with global Sustainable Development Goals, particularly Goal 3, which aims to ensure healthy lives and promote well-being for all at all ages. PPD is a rare, often misdiagnosed, yet extremely dangerous condition. WHO estimates that globally, about 10% of pregnant women and 13% of women who have just given birth experience a mental disorder, primarily depression. This includes PPD as well as other perinatal mood disorders. The American Psychological Association suggests that PPD affects approximately one in seven women in the United States [7]. However, rates may vary depending on factors such as ethnicity, socioeconomic status, and access to healthcare. In poorer nations, it is considerably more common. This is a serious public health issue that affects women and their families and significantly increases maternal mortality and morbidity [8]. In a prospective hospital-based study in Goa in 2000, among 270 mothers, depressive disorder was detected in 23% of women at six to eight weeks of delivery using the Edinburgh Postnatal Depression Scale (EPDS) [8]. In a cross-sectional community-based study conducted in rural Jharkhand and Orissa among 5,801 mothers around six weeks after delivery using the Kessler 10-item scale, 11.5% of mothers had symptoms of distress [9]. An additional community-based prospective study conducted in Vellore revealed a 19.8% prevalence of PPD [10]. The National Mental Health Survey of India (2015-2016) found that around 15% of women in India experience PPD. This prevalence may vary across different states and regions due to cultural, social, and economic factors. PPD is often misdiagnosed, despite having major repercussions and being treatable. For women with PPD, recurrent depression is another important risk factor [11]. Depression at this period of life weakens the bond with the newborn, which increases the risk of malnutrition and other issues. Neglecting an infant throughout its formative years can result in mental health issues down the road. Many moms are unaware that they are sad, while others are discouraged from getting support from others because of societal shame. If left unnoticed or found but treatment is not pursued due to a lack of knowledge about the severity of the illness, it can have detrimental effects on the mother, child, and entire family that are avoidable [9,12-14].

This study aims to estimate the prevalence of PPD and analyze sociodemographic factors associated with it among mothers in Southern Odisha. Understanding these factors is crucial for tailored interventions and can contribute to global knowledge on PPD, considering the region’s unique cultural and socioeconomic characteristics.

## Materials and methods

This population-based cross-sectional study was conducted in the rural area served by the Urban Health and Wellness Centre, Ankuli, located in the Ganjam district of Southern Odisha. Following WHO’s definition, the postpartum period, or puerperium, was considered to begin within one hour of placental delivery and extend up to six weeks (42 days) postpartum [13]. All women within the study area, regardless of residency status, who had delivered between May 2022 and November 2022 and had passed 42 days post-delivery but were within six months postpartum, were eligible for inclusion, irrespective of birth outcome. Exclusion criteria included serious illness and a lack of consent for participation in the study. Only primiparous mothers with a birth order of 1 were included. Data collection involved conducting interviews with the participants in their homes with written informed consent obtained in the native language (Odiya).

PPD data were collected using the EPDS [15], a validated screening tool known for its simplicity and effectiveness. Following content and face validity evaluation, the EPDS was translated into the native language of Odiya. The scale comprises 10 questions, each scored from 0 to 3, with a total possible score of 30. Symptoms of depression are addressed within the EPDS questions. A score of 12 or higher indicates clinically significant depressive symptoms, while a score of 10-11 suggests moderate depression. Participants were asked about experiencing specific symptoms for seven or more consecutive days during the postpartum period. Women scoring 10 or more points underwent further questioning. Approval for the study was obtained from the Institutional Ethics Committee of Maharaja Krushna Chandra Gajapati (MKCG) Medical College and Hospital, Brahmapur, Odisha, India. Following data collection and validation, the information was recorded, compiled, and subjected to analysis using JAMOVI version 2.3.18 and DATATAB. Descriptive statistics were employed to summarize demographic and clinical characteristics. The prevalence of PPD was determined, and associations with relevant factors were explored using the chi-square test, with p-values and confidence intervals calculated. Additionally, correlation analysis was performed to assess relationships between different components of the scale and sociodemographic variables.

## Results

The study’s findings reveal a diverse profile of the participants’ demographic and health characteristics (Table 1). The mean age of the mother was 26.98 ± 4 years. Regarding educational status, the majority of respondents were literate, constituting 104 cases (85.95%), while a smaller proportion, 17 cases (14.05%), were identified as illiterate. In terms of family structure, joint families were predominant, representing 86 cases (71.07%), compared to nuclear families at 35 cases (28.93%). The mode of delivery varied, with 74 cases (61.16%) opting for normal vaginal delivery (NVD) and 47 cases (38.84%) undergoing a lower uterine cesarean section (LUCS). In terms of the baby’s gender, females slightly outnumbered males, comprising 72 cases (59.50%) and 49 cases (40.50%), respectively. Notably, a small percentage, six cases (4.96%), reported a history of psychiatric illness, while the vast majority, six cases (95.04%), did not. Family and partner support were predominantly present, with 107 cases (88.43%) and 119 cases (98.35%), respectively, while adverse life events were reported by five cases (4.13%) and anemia by the same percentage.

**Table 1:** Sociodemographic profile of participants (n = 121) Adverse life events, such as financial stress, interpersonal conflict, and traumatic experiences, affect maternal mental health outcomes, positing that greater social support and resilience buffer against the adverse effects of stress. LUCS, lower uterine cesarean section; NVD, normal vaginal delivery

Sociodemographic variables	Frequency	Percentage
Illiterate	17	14.05
Literate	104	85.95
Type of family		
Joint	86	71.07
Nuclear	35	28.93
Type of delivery		
NVD	74	61.16
LUCS	47	38.84
Age of mother		
<20 years	2	1.65
20-30 years	103	85.12
30-40 years	16	13.23
Baby’s gender		
Female	72	59.5
Male	49	40.5
Past history of psychiatric illness		
Yes	6	4.96
No	115	95.04
Family support		
Yes	107	88.43
No	14	11.57
Partner support		
Yes	119	98.35
No	2	1.65
Adverse life events		
Yes	5	4.13
No	116	95.87
Anemia		
Yes	5	4.13
No	116	95.87

The results of the EPDS assessment indicate that the majority of respondents, comprising 111 cases (91.74%) of the sample, scored below the threshold of 12, suggesting a lower likelihood of experiencing postnatal depression (Table 2). Conversely, 10 cases (8.26%) of participants scored above 12 on the EPDS, indicating a higher likelihood of postnatal depression. These findings shed light on the mental health status of postpartum individuals within the study population, highlighting a notable proportion at risk of experiencing depressive symptoms.

**Table 2:** Prevalence of PPD measured on the EDPS scale (maximum score = 30) (n = 121) Responses are scored on a scale of 0 to 3 based on the seriousness of the symptom. Items 3 and 5-10 are reverse scored, with higher scores indicating lower severity. The total score is calculated by summing up the scores for each of the 10 items. Mothers scoring above 12 or 13 are likely to be suffering from depression and should seek medical attention. EDPS, Edinburgh Postnatal Depression Scale; PPD, postpartum depression

EPDS Total score	Frequency	Percentage
>=12	10	8.26
<12	111	91.74

There are significant associations between various risk factors and the prevalence of PPD (Table 3). Family type showed a significant association with PPD (p = 0.035), where women from joint families exhibited a higher prevalence of depression (10 cases) compared to those from nuclear families (zero cases). Type of delivery also emerged as a significant factor (p = 0.004), with a higher prevalence of PPD observed among women who underwent LUCS (eight cases) compared to those who had an NVD (two cases). Additionally, the baby’s gender was associated with PPD (p = 0.04), with a higher prevalence of depression observed among mothers of female infants (nine cases) compared to mothers of male infants (one case).

**Table 3:** Comparison between various sociodemographic variables and PPD (n = 121) Adverse life events, such as financial stress, interpersonal conflict, and traumatic experiences, affect maternal mental health outcomes, positing that greater social support and resilience buffer against the adverse effects of stress. The chi-square test was applied to obtain the p-value. A p-value of <0.05 is considered significant. LUCS, lower uterine cesarean section; NVD, normal vaginal delivery; PPD, postpartum depression

Sociodemographic variables	Depressed	Not depressed	P
Literacy			0.182
Literate	10	94	
Illiterate	0	17	
Family type			0.035
Joint	10	76	
Nuclear	0	35	
Type of delivery			0.004
NVD	2	73	
LUCS	8	38	
Baby’s gender			0.04
Female	9	63	
Male	1	48	
H/O psychiatric illness			0.022
No	8	107	
Yes	2	4	
Family support			0.232
Yes	10	97	
No	0	14	
Partner support			0.669
Yes	10	109	
No	0	2	
Adverse life events			0.493
No	10	106	
Yes	0	5	
Anemia			0.493
No	10	106	
Yes	0	5	

Out of the total sample of 121 mothers, 10 cases (8.26%) scored above or equal to the cutoff of 12, indicating a risk of depression. Another eight cases (6.61%) were on the borderline, scoring between 10 and 11. All the mothers with positive screening results were from a joint family, whereas none belonging to the nuclear family crossed the threshold of the screening score. Notably, most of the depressive group, eight cases (80%), were in their 20s, whereas the borderline group predominantly consisted of mothers in their 30s, accounting for six cases (75%). All the mothers in the study were from urban areas, constituting 100% of the sample, and all cases were booked under the government hospital for routine maternal care. In the depressive group, eight cases (80%) had undergone lower segment cesarean section (LSCS), while in the borderline group, 100% (eight cases) had NVD. Furthermore, only one case (10%) of the depressive group had a male infant, whereas in the borderline group, six cases (75%) had male infants.

**Figure 1:**
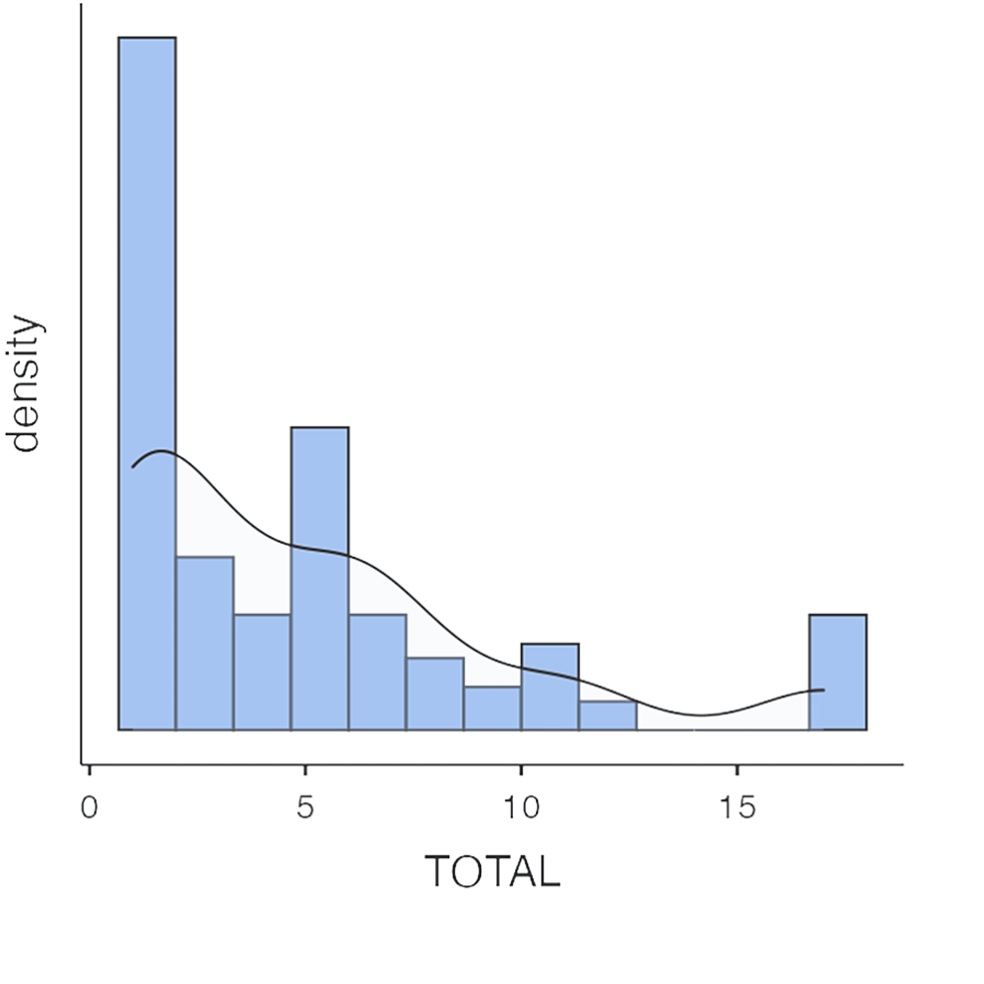
Histogram depicting the distribution of total scores Right-skewed distribution, also known as positively skewed distribution, occurs when the tail on the right side of the distribution is longer or stretched out, and the majority of the data points are concentrated on the left side. In a right-skewed distribution, the mean is typically greater than the median; as the long tail of higher values pulls the mean to the right, the data has a longer and thinner right tail, while the left tail is shorter and thicker.

The histogram (Figure 1) shows a right-skewed distribution of total scores ranging from 1 to 17, indicating that some scores are on the higher end. The substantial standard deviation of 4.45 (which exceeded the median) and variance of 19.83 highlighted considerable variability in scores, implying the presence of a subset with potentially more severe symptoms.

The point-biserial correlation analysis revealed several significant associations between individual sociodemographic variables and the total score on the scale. Firstly, there was a negative correlation between the mode of delivery (LSCS = 0, NVD = 1) and the total score, indicating that deliveries via LSCS were associated with higher total scores on the scale (rpb = -0.24, n = 121, p = 0.008). Secondly, educational status (literate = 0, illiterate = 1) exhibited a negative correlation with the total score, suggesting that literate individuals had higher scores (rpb = -0.22, n = 121, p = 0.015). Additionally, there was a negative correlation between the absence of adverse life events (no event = 0, event occurred = 1) and the total score, indicating that the absence of adverse life events was associated with higher total scores (rpb = -0.18, n = 121, p = 0.042). Similarly, the absence of anemia (no = 0, yes = 1) showed a negative correlation with the total score, suggesting that the absence of anemia was associated with higher scores on the scale (rpb = -0.18, n = 121, p = 0.042).

Table 4 revealed that while the type of family structure did not significantly impact the total score, the mode of delivery emerged as a significant predictor, indicating its notable influence even when other factors were considered. The interaction between family type and delivery mode also significantly affected the total score, emphasizing their combined effect. Additionally, maternal age demonstrated a significant influence, highlighting its role in shaping the outcome. These findings underscore the importance of the mode of delivery, maternal age, and their interaction in determining the total score while acknowledging residual variability represented by the error.

**Table 4:** ANCOVA of sociodemographic factors influencing the total score ANCOVA is a statistical technique used to compare the means of dependent variables across two or more groups while controlling for the influence of one or more continuous variables known as covariates. ANCOVA, analysis of covariance

ANCOVA	Sum of squares	df	Mean square	F	P
Type of family	6.49	1	6.49	0.39	0.531
Mode of delivery	108.57	1	108.57	6.57	0.01
Type of family * mode of delivery	177.65	1	177.65	10.74	0.001
Mother’s age	112.91	1	112.91	6.83	0.009
Error	1,918.28	116	16.54		

**Figure 2:**
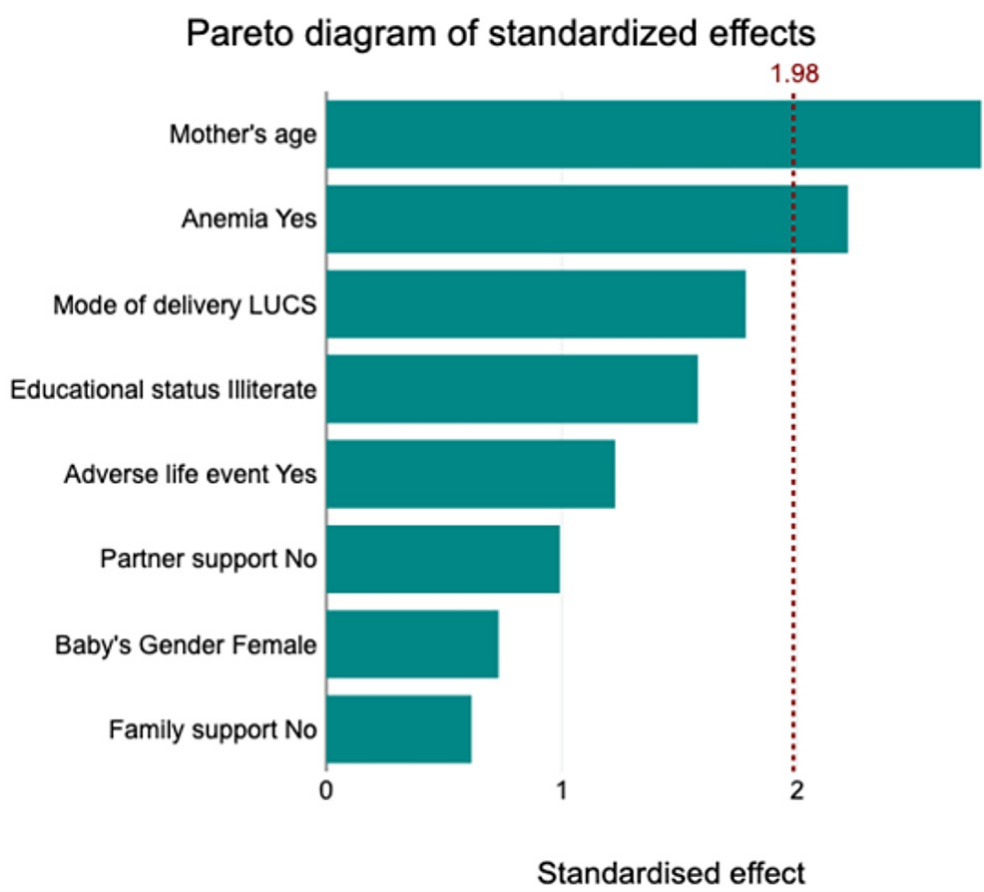
Pareto chart of the multiple factors contributing to PPD Adverse life events, such as financial stress, interpersonal conflict, and traumatic experiences, affect maternal mental health outcomes, positing that greater social support and resilience buffer against the adverse effects of stress. LUCS, lower uterine cesarean section; PPD, postpartum depression

The Pareto chart (Figure 2) indicates that the mother’s age is the most significant factor contributing to PPD, followed by anemia, mode of delivery, and educational status. Lesser, but still impactful, factors include adverse life events and a lack of partner support. The gender of the baby and family support have the least effect.

The Spearman correlation analysis explored the relationship between various aspects of the depression scale for mothers and the ages of their babies. The findings unveiled several significant associations. Firstly, there was a moderate, negative correlation between mothers feeling scared or panicky for no reason and the age of their babies (r (117) = -0.36, p < 0.001). Similarly, a moderate, negative correlation emerged between mothers experiencing anxiety or worry without reason and their child’s age (r (117) = -0.31, p = 0.001). Additionally, there was a moderate, negative correlation between mothers coping with change and the age of their babies (r (117) = -0.32, p < 0.001). Furthermore, mothers feeling so unhappy that it affected their sleep showed a low, negative correlation with their baby’s age (r (117) = -0.24, p = 0.01). However, the correlation between mothers feeling sad or miserable and their baby’s age was negligible and nonsignificant (r (117) = -0.09, p = 0.341).

## Discussion

The results of this study, which examine the connections among individual sociodemographic factors and PPD scores, provide intriguing observations, especially given certain conflicting outcomes when contrasted with existing literature. These findings challenge preconceived notions and highlight the need for diverse approaches in perinatal treatment and research, offering insightful viewpoints on the complex association between sociodemographic characteristics and PPD.

The prevalence of PPD in our study conducted in a tertiary care hospital in Southern Odisha was found to be 6.61%. This finding is consistent with the global estimate of PPD, which ranges from 10% to 15%, done by Gavin et al. (2005) [16]. It underscores the universal nature of PPD as a mental health challenge for new mothers, regardless of geographical location. Our study identified several sociodemographic risk factors associated with PPD in Southern Odisha. Mothers with higher educational attainment and income levels were more vulnerable to PPD. This observation was opposite to the findings from previous research by Gavin et al. (2005) and Gelaye et al. (2016) [16,17], raising questions about the role of socioeconomic disparities in PPD risk. Efforts to provide emotional support and mental health services to mothers with complicated pregnancies and those undergoing cesarean sections are essential. Access to healthcare services played a crucial role in PPD risk in Southern Odisha. Mothers with limited access to antenatal care and postpartum support were more likely to experience PPD. The current maternal service provision is mainly antenatal-centric, with newborn immunization being the only major postpartum programmatic component. The lack of postpartum care for the mother’s health, identification of mental stress, and supportive care despite the compulsory six postpartum visits to be conducted by the community health worker as per the government program points toward a lack of awareness and a lack of monitoring components.

The negative correlation observed between mode of delivery, specifically deliveries via LSCS, and higher total scores on the PPD scale aligns with conventional expectations [18]. This may stem from various factors, including the physical and emotional trauma associated with surgical intervention during childbirth, feelings of loss of control or disappointment regarding the birth experience, and an increased likelihood of postoperative complications and pain, as seen from the studies of Goker et al. (2012) and Andersson et al. (2004) [19,20]. Additionally, societal perceptions and expectations surrounding cesarean deliveries, such as concerns about maternal bonding and perceptions of failure to achieve a “natural” birth, may contribute to heightened psychological distress among women undergoing LSCS. Historically, natural childbirth has been associated with lower rates of PPD compared to operative deliveries, attributed to factors such as the release of endorphins during labor and greater maternal satisfaction with the birthing experience, as evident from the studies by Andersson et al. (2004) and Ayers et al. (2016) [20,21]. The clinical implications of these findings underscore the importance of comprehensive prenatal counseling and support for women undergoing LSCS, addressing potential emotional concerns, and providing tailored interventions to mitigate the risk of PPD.

The negative correlation observed between educational status and PPD scores challenges conventional assumptions regarding the protective effects of higher education against mental health disorders. The works of Goyal et al. (2010) [22] and Robertson et al. (2004) [23] associate higher educational attainment with greater access to resources, coping skills, and social support networks, which are posited to buffer against the development of PPD. However, our findings suggest a counterintuitive relationship, with literate individuals exhibiting higher PPD scores. Several hypotheses may elucidate this unexpected association. Firstly, the pressures and expectations accompanying higher education, such as career advancement, financial responsibilities, and societal norms regarding parenting and maternal roles, may contribute to heightened stress and psychological vulnerability among educated women, as evidenced by the studies by Robertson et al. (2004) [23] and Szegda et al. (2018) [24]. Additionally, the stigma surrounding mental health issues within highly educated or professional circles may deter individuals from seeking timely support or disclosing their struggles with PPD, as evidenced by the study of Szegda et al. (2018) [25]. The implications of these findings underscore the need for targeted interventions and support mechanisms tailored to the unique needs of educated women during the perinatal period. Healthcare providers should prioritize destigmatizing discussions surrounding mental health and PPD within educational settings and professional environments, facilitating access to culturally sensitive and evidence-based interventions.

The negative correlation identified between the absence of adverse life events and higher PPD scores challenges prevailing assumptions regarding the protective role of social support and stability in mitigating the risk of postpartum mental health disorders. Goyal et al. (2010) [22] and Robertson et al. (2004) [23] often emphasize the detrimental impact of adverse life events, such as financial stress, interpersonal conflict, and traumatic experiences, on maternal mental health outcomes, positing that greater social support and resilience buffer against the adverse effects of stress. However, our findings suggest a paradoxical relationship, with individuals devoid of adverse life events exhibiting higher PPD scores. This unexpected result may reflect underlying complexities in the relationship between psychosocial stressors, coping mechanisms, and resilience in the context of perinatal mental health. It is plausible that individuals with no reported adverse life events may face unique challenges or internal stressors, such as perfectionism, unrealistic expectations, or cultural norms regarding motherhood, which contribute to heightened psychological distress, as seen from the studies by Robertson et al. (2004) [23] and Szegda et al. (2018) [24]. These findings suggest the importance of nuanced assessments of psychosocial risk factors and individualized support for women during the perinatal period, irrespective of their reported absence of adverse life events.

The negative correlation observed between the absence of anemia and higher scores on the depression scale suggests a potential association between anemia and depressive symptoms. However, it is crucial to acknowledge potential confounding factors, such as blood loss during delivery, which is inevitable in all cases and may contribute to postpartum anemia. Additionally, the relationship between anemia and depression is multifaceted, with anemia possibly acting as both a cause and an effect of depression. For example, the lethargy and malaise that come with anemia may worsen the symptoms of PPD, and anemia itself may develop as a result of depression-related issues, including a low appetite or an unbalanced diet. This complex interplay warrants further investigation in future studies to better understand the mechanisms underlying the relationship between anemia and PPD. By exploring these factors more comprehensively, future research can provide valuable insights into the management and treatment of both conditions.

The findings of our study resonate with previous literature regarding the nuanced relationship between maternal depressive symptoms and the developmental stages of their infants. For instance, research by Field (2010) [25] demonstrated a decrease in feelings of fear and panic without a discernible cause as infants grow older, aligning with our observation of diminishing symptoms over time during the postpartum period. Furthermore, studies by Ross and McLean (2006) [26] have reported a decline in maternal anxiety or worry without a specific reason as the child ages, supporting our finding of decreasing maternal anxiety with the baby’s growth. Moreover, our study’s identification of a decreasing trend in coping with change as the child ages aligns with the work of Medoff-Cooper and Gennaro (1996) [27], who highlighted the challenges of postpartum adjustment and suggested that mothers may find it easier to adapt to parenthood as their baby matures. However, our observation of persistent feelings of profound unhappiness affecting sleep throughout the early stages of the child’s life echoes findings from the study by Paulson and Bazemore (2010) [28], indicating a continued struggle for some mothers despite the passage of time. Interestingly, our data also revealed stable levels of feelings of sadness or misery across the postpartum period, consistent with research by Misri et al. (2004) [29], which suggested that certain depressive symptoms may remain relatively constant over time. Overall, our study adds to the existing literature by providing insights into the dynamic nature of maternal depressive symptoms across the developmental stages of infants, emphasizing both potential improvements and persistent challenges faced by mothers during the postpartum period. Maternal health programs like SUMAN and the MAA program should integrate mental health support into routine care, ensuring early detection and intervention for PPD. Accredited Social Health Activists (ASHAs), through their compulsory home visits up to 42 days postpartum, can play a pivotal role in assessing maternal mental health, providing support, and connecting struggling mothers with appropriate resources [30]. Moreover, efforts to improve healthcare infrastructure and expand access to antenatal and postpartum care are essential for addressing maternal mental health needs effectively. Programs like LAQSHA and NQAS, while focusing on enhancing the quality of healthcare facilities, may ensure that women receive comprehensive and culturally sensitive care that encompasses mental health support [31]. Furthermore, education on cultural practices that may influence maternal mental health is crucial. Maternal health programs should incorporate culturally relevant information and interventions to address cultural factors contributing to PPD risk [32].

Strengths

The study provides valuable insights into PPD within a specific geographical context of Southern Odisha, offering region-specific data that can inform local healthcare policies and interventions. By examining a variety of risk factors associated with PPD, the study contributes to a nuanced understanding of the condition and helps identify key areas for preventive measures. The use of a cross-sectional design allows for the assessment of PPD prevalence at a single point in time, providing a snapshot of the current situation, which can be critical for timely policy and healthcare decisions. The large sample size and good representation of various sociodemographic characteristics enhance the generalizability and reliability of the findings, making the results applicable to a broader population in the region. Employing validated and standardized tools for diagnosing PPD ensures the accuracy and reliability of the data collected, increasing the credibility of the study findings.

Limitations

Reliance on self-reported measures for PPD and associated risk factors could lead to biased estimates. The regional focus on Southern Odisha may restrict the applicability of the results to other contexts. Furthermore, considering the dynamic nature of the mental health state, the single point of time data in this study does not allow the establishment of temporal relationships to prevent drawing causal inferences, while unmeasured confounders may introduce bias. Standardized screening tools may not fully capture cultural nuances, and social stigma may hinder accurate reporting. Lack of long-term follow-up and a focus on a subset of risk factors may limit comprehensive understanding and preventive strategies for PPD.

Recommendations

These limitations highlight the complexity of maternal mental health and underscore the need for holistic approaches in research and care practices. To address the significant findings regarding risk factors for PPD, proactive measures can be implemented. In the first place, early identification of at-risk mothers during booking and antenatal check-ups is crucial. Healthcare providers should conduct comprehensive assessments to identify risk factors such as a history of psychiatric illness, a lack of family support, and adverse life events. In the second place, tailored counseling sessions for at-risk mothers during antenatal care visits can prevent PPD onset by providing emotional support, coping strategies, and self-care education. Additionally, implementing active screening for PPD by ASHAs during routine home visits using standardized tools like the EPDS can facilitate early detection and intervention. Integrating active screening programs for PPD within healthcare facilities and providing a supportive atmosphere for mothers with symptoms of post-traumatic stress disorder is essential. These strategies aim to enhance early detection, prevention, and management of PPD, ultimately improving maternal and child health outcomes.

In subsequent studies, it is imperative to delve into nuanced aspects influencing the connection between the mode of delivery and PPD, such as maternal perspectives, psychosocial backing, and post-surgical encounters. Additionally, there is a need for research to investigate the interaction between educational attainment, sociocultural elements, and psychological wellness to unravel the intricacies of this association. Furthermore, exploring the interplay of subjective stress experiences, coping mechanisms, and cultural influences in shaping maternal mental health outcomes is essential for guiding tailored interventions and policy formulations.

## Conclusions

This study emphasizes the multifaceted nature of PPD and its associated risk factors among mothers in Southern Odisha. Significant associations were observed between PPD and factors such as a history of psychiatric illness, a lack of family and partner support, adverse life events, and anemia. In particular, maternal age emerged as a prominent predictor of PPD, alongside surgical mode of delivery, higher educational status, and the absence of adverse life events. The analysis of covariance further highlighted the interactive influence of joint family and delivery mode on PPD scores, emphasizing the importance of considering sociodemographic factors in understanding maternal mental health outcomes. The Pareto chart delineated the relative contributions of various factors to PPD, with maternal age and health-related factors exerting the most significant impact. Additionally, the Spearman correlation analysis revealed nuanced associations between depressive symptoms and the ages of infants, suggesting evolving maternal experiences throughout the postpartum period. These findings show the complexity of PPD and emphasize the need for comprehensive interventions targeting sociodemographic, health-related, and psychosocial factors to support maternal well-being in Southern Odisha and beyond.
